# Epidemiology of and risk factors for neonatal candidemia at a tertiary care hospital in western China

**DOI:** 10.1186/s12879-016-2042-9

**Published:** 2016-11-24

**Authors:** Jichang Chen, Yongjiang Jiang, Ba Wei, Yanling Ding, Shaolin Xu, Peixu Qin, Jinjian Fu

**Affiliations:** Department of Neonatology, Liuzhou Maternity and Child Health Care Hospital, Liuzhou, China 545001

**Keywords:** Neonate, Candidemia, Case-control study, Risk factors

## Abstract

**Background:**

The prevalence and clinical characteristics of neonatal candidemia are poorly understood in western China. The aim of our study was to evaluate the epidemiological features of neonatal candidemia in the Liuzhou Maternity and Child Healthcare Hospital.

**Methods:**

A retrospective case-control study was conducted between January 2012 and November 2015. Electronic databases were reviewed and data on *Candida* species were isolated from blood cultures and candidemia incidence, risk factors, and mortality were extracted. Univariate and multivariate logistic regression analysis were performed to identify risk factors associated with candidemia.

**Results:**

During the 4-year period, candidemia was identified in 69 newborns, for an incidence rate of 13.6 per 1000 admissions. Prolonged antibiotic therapy duration [odds ratio (OR), 95% confidence incidence (95% CI) = 1.06, 1.01–1.10], total parenteral nutrition [OR, 95% CI = 6.03, 2.10–17.30] and neurodevelopmental impairment (OR, 95% CI = 7.34, 1.18–45.80) were all associated with increased odds of candidemia development in infants (*P* value was 0.010, 0.001, 0.033, respectively). The overall mortality rate was 7.2% in the candidemia group.

**Conclusions:**

Prolonged duration of antibiotic therapy, presence of total parenteral nutrition and neurodevelopmental impairment were the major risk factors associated with neonatal candidemia. This study highlights the importance of the early detection, diagnosis and treatment of neonatal candidemia.

## Background

*Candidiasis* is one of the leading causes of bloodstream infections in neonatal intensive care units (NICUs) and associated with high morbidity and mortality. It has been estimated that 2.4–9.0% of mortality [1, 2] and 25.0% of morbidity [3] in the NICU setting may be attributable to *Candida* infections.

Because of their immature specific and nonspecific immune systems, neonates may be vulnerable to invasive candidemia. Several factors have previously been identified as contributing to an increased incidence of neonatal candidemia, including prolonged endotracheal intubation, indwelling medical catheterization, parenteral nutrition, use of broad-spectrum antibiotics, and prolonged antibiotic therapy duration. Because of the lack of specific signs and symptoms and sensitive and specific laboratory tests for the diagnosis of *Candida* infection, early diagnosis of candidemia remains crucial and is a challenge for both pediatricians and microbiologists. Therefore, the aims of the present study were to evaluate the incidence and epidemiology of candidemia in infants and determine the risk factors and clinical outcomes associated with candidemia to help pediatricians select effective preventive measures and medical treatment for neonatal candidemia.

## Methods

This retrospective study was conducted at the 60-bed NICU of the Liuzhou Maternity and Child Healthcare Hospital, which is the largest neonatal care center in Liuzhou. Between 1000 and 1200 neonates are admitted to this NICU each year. Candidemia was defined by a blood culture that yielded any *Candida* species. Microbiology laboratory and clinical records from two electronic databases were reviewed. The following data were extracted: admission age, gender, gestational age, birth weight, delivery mode, necrotizing enterocolitis, neurodevelopmental impairment, maternal underlying diseases, abdominal surgery, mechanical ventilation, indwelling central venous catheterization, endotracheal intubation, rescue history, total parenteral nutrition, hospitalization duration, use of carbapenems, use of vancomycin, multiple antibiotic (≥3 classes of antibiotics) use, antibiotic therapy duration and outcome of candidemia. For each case, one neonate with negative blood culture results was matched based on the following factors to serve as a control: admission age, gender, gestational age, and birth weight. These data were also extracted as previously described.

### Microbiologic methods

All microbiological testing was completed using standard methodology. *Candida* were isolated from blood cultures using the BacT/AlerT 3D rapid culture and monitoring system (BioMérieux). *Candida* species were identified using API 20C AuX (BioMérieux).

### Statistical analysis

SPSS version 20.0 (SPSS Inc. Chicago, Il, USA) was used for data analysis. Comparisons between the case and control groups were performed using univariate analysis methods. All variables with *P* < 0.05 were selected for inclusion in the multivariate logistic regression model to identify predisposing risk factors associated with neonatal candidemia.

### Ethical considerations

Local ethics approval was obtained.

## Results

Five thousand and seventy-five newborns were admitted to the NICU from January 2012 to November 2015, and 69 newborns developed candidemia. In the case group, the gestational age ranged from 27 weeks to 41 weeks and birth weight ranged from 800 g to 3350 g. Of the 33 infants with extremely low birth weight (ELBW < 1000 g), 10 developed candidemia; of the 449 newborns were very low birth weight (VLBW < 1500 g), 38 developed candidemia. The overall candidemia incidence was 13.6 per 1000 admissions. The highest candidemia incidence was observed among ELBW infants (303.0 per 1000 ELBW infants). The candidemia incidence among VLBW infants was 84.6 per 1000 VLBW infants. The overall mortality rate in the candidemia group was 7.2%, while the mortality rate in the control group was 1.4%.

The most and almost equally prevalent pathogens identified were *Candida albicans* and *Candida glabrata*, with 30 (34.5) and 23 (33.3%) episodes of candidemia attributed to *Candida albicans* and *Candida glabrata*, respectively. The remaining episodes of candidemia were caused by *Candida tropicalis* (14, 20.3%), *Candida parapsilosis* (1, 1.4%) and *Candida kefyr* (1, 1.4%).

Patient demographics, clinical characteristics, and prognosis were compared between the candidemia and non-candidemia groups and are summarized in Table 1. There were no significant differences in the following variables between case and control patients: gestational age, birth weight, admission age, gender and delivery mode.

**Table 1:** Clinical characteristics of neonates with and without candidemia

Variable	Case mean (95% CI) or *n*(%)	Control mean (95% CI) or *n*(%)	*P* value	Odds ratio (OR) (95% CI)
Demographics				
Gestational age (wks)	31.7(28.2, 35.2)	31.6(27.8, 35.4)	0.763	
Birth weight (g)	1514.4(852.0,2176.3)	1529.3(856.5,2202.1)	0.894	
Male gender, n(%)	44(63.8)	41(59.4)	0.600	1.20(0.61–2.39)
Admission age	1.1(0.8, 1.4)	1.5(1.2, 4.2)	0.149	
Risk factors				
Vaginal delivery	28(40.6)	33(47.8)	0.392	0.75(0.38–1.46)
Necrotizing enterocolitis	16(23.2)	6(8.7)	0.025	3.17(1.16–8.68)
Neurodevelopmental impairment	14(20.3)	2(2.9)	0.006	8.52(1.86–39.14)
Maternal underlying diseases	31(44.9)	19(27.5)	0.035	2.15(1.06–4.37)
Abdominal surgery	10(14.5)	3(4.4)	0.057	3.67(0.96–13.99)
Mechanical ventilation	43(62.3)	28(40.6)	0.011	2.42(1.22–4.80)
Central venous catheter	40(58.0)	24(34.8)	0.007	2.59(1.30–5.15)
Intubation	31(44.9)	17(24.6)	0.013	2.50(1.21–5.15)
Rescue history	35(50.7)	26(37.7)	0.124	1.70(0.86–3.35)
Total parenteral nutrition	58(84.1)	23(33.3)	0.000	10.55(4.66–23.85)
Hospitalization duration (d)	46.6(23.3, 69.9)	24.5(5.7, 43.3)	0.000	
3^rd^ cephalosporins use	39(57.4)	29(42.0)	0.074	1.86(0.94–3.65)
Carbapenems use	50(72.5)	15(21.7)	0.000	9.47(4.35–20.64)
Vancomycin use	10(14.5)	5(7.2)	0.179	12.17(0.70–6.72)
Multiple antibiotic use	37(53.6)	9(13.0)	0.000	7.71(3.31–17.95)
Antibiotic therapeutic duration (d)	33.5(14.7, 52.3)	13.8(2.5, 25.1)	0.000	
Outcome				
Cure	16(23.2)	32(46.4)	reference	reference
Improvement	43(62.3)	33(47.8)	0.013	2.61(1.23–5.53)
Exacerbation	5(7.2)	3(4.3)	0.128	3.33(0.71–15.74)
Death	5(7.2)	1(1.4)	0.043	10.00(1.08–92.94)

In the univariate logistic regression analyses, factors significantly associated with candidemia were necrotizing enterocolitis (*P* = 0.025), neurodevelopmental impairment (*P* = 0.006), maternal underlying diseases (*P* = 0.035), mechanical ventilation (*P* = 0.011), central venous catheterization (*P* = 0.007), intubation (*P* = 0.013), total parenteral nutrition (*P* = 0.000), prolonged hospitalization duration (*P* = 0.000), carbapenem use (*P* = 0.000), multiple antibiotic use (*P* = 0.000), antibiotic therapy duration (*P* = 0.000), and the mortality due to candidemia (*P* = 0.043).

Forward step-wise multivariate logistic regression was used to evaluate the risk factors for candidemia identified as significant in the univariate analyses, as shown in Table 2. The results of this analysis showed that total parenteral nutrition [odds ratio (OR) = 6.03, 95% confidence interval (95% CI = 2.10–17.30, *P* = 0.001], antibiotic therapy duration (OR = 1.06, 95% CI = 1.01–1.10, *P* = 0.010), and neurodevelopmental impairment (OR = 7.34, 95% CI = 1.18–45.80, *P* = 0.033) were significant predictors of candidemia in the multivariate model.

**Table 2:** Multivariate analysis for candidemia

Risk factor	Odds ratio	95% CI	*P* value
Total parenteral nutrition	6.03	2.10–17.30	0.001
Antibiotic therapeutic duration (d)	1.06	1.01–1.10	0.010
Neurodevelopmental impairment	7.34	1.18–45.80	0.033

## Discussion

*Candida* species have emerged as a leading pathogenic cause of bloodstream infections in neonates [1–3]. The overall incidence of candidemia in our study was 13.6 per 1000 admissions. This incidence was lower than that reported in a previous study conducted by Benjamin et al. [1], in which a higher incidence (9%) was reported. In the present study, we found that the overall incidence of candidemia among VLBW infants was 84.6 per 1000 admissions, and in the ELBW group, the candidemia incidence was 303.0 per 1000 admissions. These findings are consistent with the results of a study based on surveillance data collected in England between 2004 and 2010, which reported that ELBW infants had the highest risk of invasive *Candida* infection of the neonates evaluated [2]. During the 4-year period evaluated in our study, only 33 ELBW infants survived and were admitted to the NICU after delivery. One likely reason for the observed discrepancy is that the prevalence of VLBW, especially ELBW, among infants is much higher in developed countries than in China [4]. These studies highlighted the importance of infection control and early identification of potential risk factors for the prevention of candidemia among high risk infants.

In our study, the majority of candidemia episodes were caused by *C. albicans* (43.5%) and *C. glabrata* (33.3%). Previous studies, especially those conducted in western countries, have frequently reported that *C. albicans* was the most common causative agent of neonatal candidemia, followed by *C. parapsilosis* [2]. In contrast with the results of some previous studies, the results of our study paralleled those of a previous study conducted by Xia et al. [4], who reported that *C. albicans* was the species most commonly identified in Chinese 11 NICU centers, followed by *C. glabrata*. These results were consistent with the fact that the predominant causative agents of candidemia may vary by geographic region [5].

Previous studies have revealed that prophylactic or empiric therapy with antifungal agents, especially fluconazole, may be associated with changes in *Candida* ecology and antifungal agent susceptibility [6]. According to the microbiological data evaluated in our study, all of the tested *Candida* isolates were susceptible to fluconazole, amphotericin B and voriconazole. Fluconazole is considered as the first line antifungal agent in our hospital. Previously, a study showed that pre-exposure to fluconazole was a predisposing factor for *C. glabrata* infection [7], which may partly explain the observed discrepancy regarding the distribution of *Candida* among neonates, as most NICUs have been reported to use liposomal amphotericin B when administering prophylactic or empiric therapy [2].

Central venous catheterization and total parenteral nutrition were identified as significant predisposing factors for the development of candidemia in our study. The majority of the infected neonates had received central venous catheterization (58.0%), endotracheal intubation (44.9%), and total parenteral nutrition (84.1%). The multivariate logistic regression model results suggested that total parenteral nutrition was the factor most highly associated with increased odds of candidemia. *Candida* species are notorious for their capacity to attach to foreign materials (such as invasive and indwelling devices) and form biofilms, which may be associated with high virulence and act as a biological barrier that prevents the penetration of antifungal agents and protects the fungal cells from the host’s immune responses. This fact may explain why invasive and indwelling medical devices have been identified as factors consistently associated with increased risk of candidemia [5, 8].

Broad-spectrum antibiotic use has also been described as a risk factor for candidemia [2, 9, 10]. In our department, the majority of candidemia cases had received broad-spectrum antibiotics, such as carbapenems (72.5), and 53.6% of patients received multiple antibiotics. The median antibiotic therapy duration was 33.5 days (range 14.7–52.3 days). A previous study conducted by Kaufman et al. [11] showed that decreased use of carbapenem may be associated with decreased incidence of invasive fungal infections. It has been reported that prolonged exposure to broad-spectrum antibiotics not only increases the risk of developing neonatal candidemia [12] but also may be associated with the development of refractory candidemia [13]. The widespread use of antibacterial agents may suppress bacterial flora and increase *Candida* colonization density [14]. The results of our study were consistent with those of a previous investigation conducted in 11 NICUs, which revealed that broad-spectrum antibacterial agents were commonly used for prophylactic or empiric therapy in China [4]. This finding highlights the need to evaluate the antimicrobial burden in local NICUs in China.

Our study revealed that neonatal candidemia was associated with neurodevelopmental impairment. Several studies have also reported this conclusion. Two large prospective cohort studies reported that systemic candidiasis was associated with increased risk of death and/or neurodevelopmental impairment [15, 16] in extremely low birth weight infants. Experimental data suggests that the cytokine-mediated inflammatory responses mediated elicited by infection may be neurotoxic and contribute to brain damage. However, data regarding cytokine-mediated inflammatory responses to specific pathogens and their relationship with adverse neurodevelopmental outcomes are limited [17, 18]. It is of importance to evaluate potential mechanisms by which to reduce inflammatory responses and the risk of brain damage associated with candidemia in future research.

As was identified in mainland China, the situation related to neonatal candidemia observed in this study was also not optimistic. Studies have previously reported incidence rates of candidemia ranging from 0.74 [4] to 15.7% [19] and mortality rates ranging from 8.9 [20] to 26.3% [21] in neonatal patients in mainland China. Our results were consistent with those of previous investigations [4, 19, 22, 23] suggesting that very low birth weight was an independent factor associated with the development candidemia. Previous studies have identified *C. albicans* as the predominant pathogen causing candidemia in neonatal patients in China [4, 22]. Risk factors previously reported to be associated with neonatal candidemia in China have included intubation [20], use of medical catheters [20, 21, 23], use of high-level antibiotics [20, 21], prior surgeries [20, 21], prolonged antibiotic use [21], and preterm birth with low birth weight [4, 19, 22, 23].

The limitations of the current study were inherent given its retrospective nature. The single center design and small sample size may have compromised the statistical power of the study. Furthermore, the diagnosis of neurodevelopmental impairment was mostly based on ophthalmological, otological, and radiological findings and not systematically evaluated using the Gross Motor Functional Classification Score and cognitive and motor scales of the Bayley Scales of Infant Development-III (BSID-III) [16]. Follow-up data regarding neuro- developmental impairment in the infants included in this study were not available. Further studies should focus on this aspect of candidemia risk. Nevertheless, these data provide information regarding the epidemiology of neonatal candidemia in western China. It is crucial for local pediatricians to promote the prevention, early detection and treatment of candidemia in high risk neonates.

## Conclusions

In conclusion, our findings suggest that prolonged antibiotic therapy duration, presence of total parenteral nutrition and neurodevelopmental impairment were associated with increased odds of neonatal candidemia. The identification of risk factors associated with increased odds of neonatal candidemia emphasizes the need for early detection, diagnosis and treatment of *Candidiasis* infections in NICUs.

## Abbreviations

95% CI: 95% confidence interval
BSID-III: Bayley scales of infant development-III
*C. Albicans*: *Candida albicans*
*C. Glabrata*: *Candida glabrata*
*C. Parapsilosis*: *Candida Parapsilosis*
ELBW: Extremely low birth weight
NICUs: Neonatal intensive care units
OR: Odds ratio
VLBW: Very low birth weight
